# Health Research Using Facebook to Identify and Recruit Pregnant Women Who Use Electronic Cigarettes: Internet-Based Nonrandomized Pilot Study

**DOI:** 10.2196/12444

**Published:** 2019-10-18

**Authors:** Harold H Lee, Yuli Patrick Hsieh, Joe Murphy, Jennifer W Tidey, David A Savitz

**Affiliations:** 1 Department of Social and Behavioral Sciences Harvard TH Chan School of Public Health Boston, MA United States; 2 RTI International Research Triangle Park, NC United States; 3 Department of Behavioral and Social Sciences Brown School of Public Health Providence, RI United States; 4 Department of Epidemiology Brown School of Public Health Providence, RI United States

**Keywords:** e-cigarette, pregnancy, social media

## Abstract

**Background:**

Participant recruitment is often a challenge, particularly enrolling individuals with relatively rare characteristics. The wide reach of social media may provide a mechanism to overcome these challenges.

**Objective:**

This paper aimed to provide information to researchers who seek to recruit participants from rare populations using social media for studies with demanding protocols. We aimed to describe a pilot study protocol that identified and enrolled pregnant women (second or third trimester) who were exclusive users of electronic cigarettes (e-cigarettes). We have described the recruitment methods, time, and cost; examined advertisement types that were more or less successful; discussed participant retention and relationship management; and described the process of collecting biological data.

**Methods:**

In an open-access, nonrandomized pilot study, we placed Facebook advertisements that were selectively targeting women who were likely to be pregnant and interested in e-cigarettes or vaping. The advertisements invited individuals to complete a fully automated eligibility screener based on Qualtrics. Eligible participants were asked to (1) complete a Web-based survey that collected detailed information on the use of e-cigarettes, including the exact type of device and electronic liquid, (2) report the frequency and intensity of e-cigarette use for 3 months before pregnancy and during each trimester, and (3) provide a saliva specimen for a nicotine biomarker assay. We collected a photograph of each participant’s e-cigarette device, 8 weeks after the mother’s due date, to allow corroboration of the self-report and the baby’s birth weight and gestational age from the participant’s physician.

**Results:**

Participants were recruited between August 19 and October 26, 2017. We enrolled 20 participants in 2 months at a cost of US $3421.28. Baseline data were collected for all 20 participants. Of the 20 women enrolled, 16 provided a saliva sample, 4 provided a photo of the e-cigarette device, and 10 provided physician contact information. Of the 10 physicians contacted by mail, 6 responded with information on the participants and their babies.

**Conclusions:**

Study findings suggest that Facebook’s targeting criteria should focus on e-cigarette users to maximize advertisement exposure of potentially eligible women. In addition, saliva sample collection was feasible among pregnant women (second or third trimester) who were exclusive e-cigarette users, but obtaining photographs and physician reports was problematic and called for further refinement. These lessons are likely useful to others who are seeking to use social media to recruit participants from rare populations into studies with demanding protocols.

## Introduction

### Background

Participant recruitment is a challenge in public health research, particularly when the study aims to enroll those who are difficult to reach or recruit into research studies [1,2]. The population of interest may be rare among the overall population (eg, cystic fibrosis, Huntington disease, or Duchenne muscular dystrophy), difficult to identify or contact (eg, sex workers), or hesitant to participate because of stigma or legality (eg, people with substance use disorders, abuse victims, or illegal immigrants) [3-5]. To obtain a sufficient number of hard-to-reach participants, a researcher could conduct multicenter trials, but this can be quite expensive. An approach that has been increasingly used to address this challenge is to utilize social media to recruit from the larger population.

*Social recruitment* of study participants using Facebook has gained significant traction in recent years [6-8]. According to Pew Research Center’s survey in 2018, about 80% of US adults aged 18 to 49 years use Facebook [9]. Facebook enables researchers to place targeted advertisements to reach populations with certain sociodemographic attributes (eg, age, sex, ethnicity, and location) and who communicate about their interests in a manner that makes them most likely to have the specific attributes needed for a given study. In addition, the platform enables individuals to enroll in a study by simply clicking on an advertisement and then being directed to the study site. Facebook recruitment has been successfully employed in recruiting tobacco users [10], substance abusers [6], and those suffering from depression [7]. The nascent literature on recruitment of electronic cigarette (e-cigarette) users using social media (eg, Facebook and Twitter), conducted by our team [11] and others [12], has shown promising results. However, the previous research solely relied on self-report measures to assess relevant variables (eg, e-cigarette usage and pregnancy status). A more rigorous approach requires corroboration of survey reports with objective evidence.

### Objectives

Thus, the aim of this study was to examine the utility of Facebook to recruit pregnant women who used e-cigarettes into a study with a protocol that required acquisition of saliva samples, extensive surveys assessing details of e-cigarette use (20-30 min), photographs of the e-cigarette device, and physician corroboration regarding the pregnancy and infant. This pilot study was intended to inform us for developing a large study (>1000 pregnant e-cigarette users) to evaluate the effect of pregnant mothers’ e-cigarette usage on infant health outcomes, primarily birth weight and risk of preterm birth. One predictable challenge was the ability to identify and recruit a sufficient number of participants. According to nationally representative data on both tobacco products and e-cigarette use during pregnancy, 4.9% of pregnant women were estimated to have used e-cigarettes from 2013 to 2014 [13]. The prevalence of e-cigarette use was much greater among current tobacco smokers (28.5%) but also included a sizable proportion of former smokers who switched to become exclusive e-cigarette users (prevalence of 2.3%). Thus, enrolling over 1000 pregnant women who used e-cigarettes exclusively and not in addition to tobacco—that is, minimum power to detect a potentially subtle effect of e-cigarette use on prenatal health for the larger study that will be informed by this pilot study—is unlikely to be feasible when the study is conducted at a single research site. Besides, recruiting pregnant women who use e-cigarettes into research studies may be difficult because of the stigma associated with risky behaviors during pregnancy, which can be reduced when the study is primarily conducted on the Web without the need to communicate directly with an interviewer and remain somewhat anonymous. These factors motivated this study to test the feasibility of employing Facebook recruitment. We conducted a pilot study to develop methods for an internet-based study of the effect of e-cigarettes on pregnancy outcomes, requiring recruitment of exclusive e-cigarette users in the second or third trimester of pregnancy. This study reports on recruitment methods, time, and cost, examines advertisement types that were more or less successful, discusses participant retention and relationship management, and describes the process of collecting biospecimens from participants. The lessons learned from our experience are likely useful to others who are seeking to use social media to recruit populations that are difficult to reach for studies with equally demanding protocols.

## Methods

### Ethics Approval and Consent to Participate

The Institutional Review Board of Brown University has approved the procedures presented in this paper. All participants were required to provide informed consent to participate in this study.

### Overview

This study was an open-access (ie, participants self-enroll), nonrandomized pilot study. With regard to human involvement, advertisement, screening, and enrollment in the study was purely Web-based, and once enrolled, the research assistant contacted the participants only via email and there were no on-site visits. We used Facebook to recruit 20 women who were in their second or third trimester of pregnancy and used e-cigarettes but not tobacco. Our initial plan was to enroll 30 participants and determine how many we were able to retain throughout the study. Participants were recruited between August 19 and October 26, 2017. The recruitment ceased after exhausting the allocated predetermined budget. To our knowledge, there were no critical secular events (eg, significant changes in the available internet resources) during this period. The Facebook advertising tool placed messages in their feed selectively to target women who were likely to be pregnant and interested in e-cigarettes or vaping. The advertisement invited them to complete a Web-based eligibility-screening questionnaire. Inclusion criteria for this study were as follows: (1) pregnant women who were in their second or third trimester, (2) aged 18 to 50 years, (3) currently a nonsmoker of tobacco, and (4) an e-cigarette user (≥5 days per week). The 5-day cut off point for the e-cigarette user was selected to minimize the variation in e-cigarette usage and in an effort to ensure that it was prevalent enough to have a meaningful effect on typical nicotine levels. After completing a screening survey, eligible participants were asked to read the consent form and complete the baseline questionnaire. Finally, each participant was asked to provide contact information to enroll in the study.

The survey instrument collected detailed information on (1) the exact type of e-cigarette device and electronic liquid used and (2) the frequency and intensity of use for 3 months before pregnancy and during each trimester of pregnancy. We requested a photograph of each participant’s e-cigarette device, 8 weeks after the baby’s due date, to allow corroboration of the self-report and the baby’s birth weight and gestational age from the mother’s physician.

### Facebook Recruitment

To identify and recruit currently pregnant women who use e-cigarettes exclusively, we employed Facebook’s advertisement tool from its suite of business user services. One of the advantages of recruiting on Facebook or other social media platforms is that the platform uses a *pixel* code—a short programming script that is placed on an advertiser’s website to track traffic conversions from the platform’s advertisements [14]. The pixel code allows Facebook’s algorithm to characterize users who interact with an advertisement (eg, like, share, or directly click the post) and then further disseminate the advertisement to other Facebook users with similar characteristics. To ensure tracking all conversions with the Facebook pixel in advertisements, (1) click *Show Advanced Options* under Ads in Ads Manager; (2) check *Track all conversions from my Facebook pixel*; (3) click Save and Close; (4) apply to all campaigns if not default tracking with Facebook pixel.

We first used a *conversion tracking* advertisement and later used a *traffic tracking* advertisement [15,16]. Although both methods use Facebook pixel to gather information from Facebook users, they target slightly different groups. Conversion tracking gathers information from only those who end up at the final Web page (eg, those who purchase an item, sign up for a study, or provide contact information). On the other hand, traffic tracking gathers information from those who click through the processes (eg, an advertisement, screener, study explanation, or baseline questionnaire). As an analogy, on a clothing store website, conversion tracking would collect customer information from only those who purchased clothing, whereas traffic tracking would collect customer information from those who did not purchase any clothing but showed significant interest in certain items such as viewing it or adding it to their cart.

**Figure 1:**
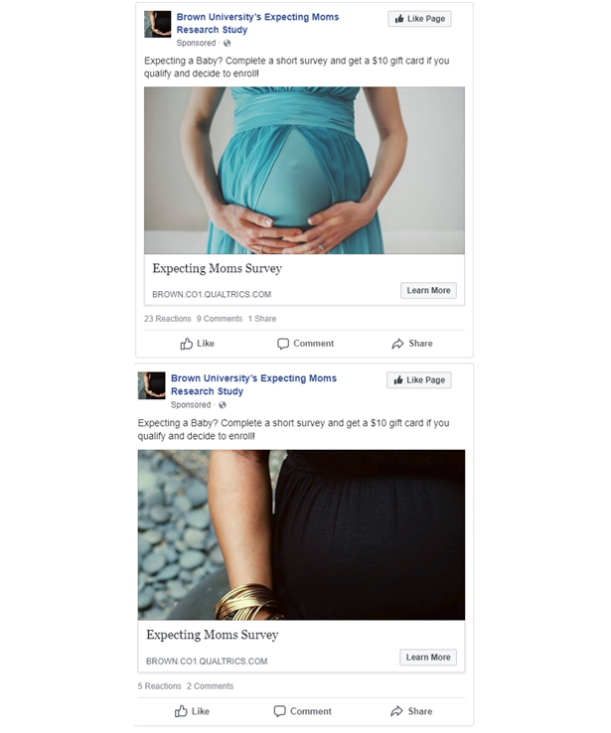
Facebook advertisements.

In this study, this screening process—from clicking the Facebook advertisement to providing contact information to complete enrollment in the study—took about 20 to 30 min. In this case, conversion tracking gathered information only from Facebook users who provided their contact information on the last page. Traffic tracking, on the contrary, allowed the algorithm to use specific demographic characteristics of Facebook users who spent a significant amount of time on activities leading up to the final Web page even if they did not complete the enrollment process. For the first 8 advertisements shown in Figure 1, we used conversion tracking that was used successfully by our group in the past [11] but was not as successful in another study recruiting smokers [10].

After employing the 8 advertisement sets (Ad sets) using conversion tracking, we contacted the Facebook marketing consultant (known as a marketing expert) because of the low screening rate. Through phone meetings and emails, the Facebook consultant helped us understand how to optimize current campaigns, but this service was provided only for a 28-day period. The consultant suggested we use traffic tracking instead of conversion tracking. The suggestion was motivated by the fact that the screening process of this study is similar to a process (ie, ideal for traffic tracking) than to a snapshot decision (ie, ideal for conversion tracking). An integral metric to determine the cost-effectiveness of conversion versus traffic is *cost per results*, which can be found in the summary table provided in Facebook Ads Manager. Cost per results counts the number of results (eg, conversion or traffic) a researcher received based on the settings for the advertisement. For example, if a researcher selected conversions as their campaign objective, the results metric may show the number of *View Contents* that occurred because of someone seeing their advertisement. In this study, for conversion, the cost per results was *View Contents*. For traffic, the cost per result was *Link Clicks*.

### Data Collection From Participants

After clicking on the advertisement, participants were first screened to see if they were eligible for the study. Eligible participants were then asked to do the following:

1. Complete an enrollment questionnaire on the Web that included their demographic background, history of e-cigarette and tobacco use, and current e-cigarette use.
2. Complete a detailed 3-day diary of e-cigarette use, receive a saliva collection kit via mail, and mail back a saliva sample that was assayed for the nicotine metabolite, cotinine, as an indicator of the level of recent nicotine exposure.
3. Provide pictures of the e-cigarette device by uploading digital photos via a Dropbox link embedded in the survey.
4. After giving birth, complete a follow-up questionnaire to update e-cigarette use information for the later period of pregnancy and report on the outcome of the pregnancy.
5. Grant permission to obtain the information on the birth outcome from the participant’s doctor to verify the birth weight and gestational age.

All surveys were conducted on the Web using the Qualtrics platform [17]. To compensate participants for their time, we provided Amazon’s electronic gift cards as incentives at each stage of the process: enrollment (US $10), completing the 3-day e-cigarette diary and providing a saliva specimen (US $25), completing the follow-up questionnaire and providing consent to review birth records after delivery (US $10), and a bonus for those who completed all 3 parts (US $15). We did not specify a period for participants in which they should submit the aforementioned data to obtain incentives. Safety and security procedures were elaborated in the informed consent form, which was collected on the Web via Qualtrics. With regard to harms or unintended effects, we noted the following:

the only risk to you of participating would be if personal information about you or your baby reached people other than the researchers. We will be extremely careful to do everything we can to make sure that doesn’t happen. Your data will be stored on a computer at the Brown University, never on a laptop or memory stick. The data will only be seen by people on the research team who need to work with it or the Brown Institutional Review Board overseeing the study. There are no direct benefits to you from being in the study. However, you may be contributing to research that will help us provide more accurate information about e-cigarettes and health to expectant mothers in the future.

### Data Collection From Physicians

Although self-report is known to be accurate for birth weight and very good (but not perfect) for gestational age [18], it is important to maximize accuracy, as we were interested in potentially subtle effects of e-cigarette use on pregnancy outcome. Birth records use both the last normal menstrual period and a clinical estimate as a means of assigning gestational age at delivery. As the clinical estimate reflects all the information available to the clinician, most importantly ultrasound, it has been shown to be the more accurate approach than relying on reporting of the last menstrual period to calculate gestational age at delivery [19]. Although birth records are a more accurate measure, whether it can be obtained successfully in clinical trial is unknown, and thus, this study attempted to test its feasibility. Toward this end, we contacted participants and asked permission to obtain birth outcome information from their doctors 8 weeks after the baby’s due date. Then, we contacted physicians to request the baby’s health information by mail or fax. Physicians were informed that the mother had provided written consent to share this information. To facilitate the process, we inserted a preaddressed envelope with a stamp. If a physician did not reply within 2 weeks, research staff contacted the physician’s office via phone.

## Results

### Facebook Recruitment

Using Facebook, we were able to identify and enroll 20 participants in a 2-month period. Using conversion tracking, we launched 8 Ad sets sequentially from August 19 to September 13, 2017 (Table 1).

**Table 1:** Conversion per Facebook advertisement specifications and enrollment outcome.

Advertisement set (Ad set)	Daily spend (US $)	Duration (days)	Number of advertisements	Conversion window (day)	Total cost (US $)	Total enrolled	Total screened
Ad set 1	200.00	6	3	7	829.86	5	306
Ad set 2	200.00	3	3	7	162.52	1	51
Ad set 3	200.00	3	2	7	741.66	5	228
Ad set 4	200.00	3	3	7	599.32	4	51
Ad set 5	300.00	3	3	7	598.93	3	170
Ad set 6	150.00	3	1	1	448.91	0	89
Ad set 7	150.00	2	1	1	298.60	0	82
Ad set 8	150.00	2	1	1	299.95	1	172

We incrementally fine-tuned our parameters for the subsequent set based on the turnout of the ongoing set. The specifics of daily budget and audience details were informed by our team’s previous research [11]. Ad sets 1 and 2 used the same search terms, which included *electronic cigarettes*, *vapor*, and *pregnancy*, and enrolled 5 participants. As many people were screening out from Ad set 1, we added *baby shower* and *new moms* to the existing search terms in Ad sets 3 and 4 and enrolled an additional 9 participants. This may have influenced the reduction in per enrollment cost from approximately US $165 (Ad sets 1 and 2) to US $150 (Ad sets 3 and 4). In Ad set 5, we increased the daily money spent to US $300 (from US $200) and enrolled an additional 3 participants, but this resulted in a higher per enrollment cost of approximately US $200. In Ad set 6, daily money spent was reduced to US $150; the number of advertisements within an Ad set was reduced from 3 to 1; and conversion window was reduced to 1 day from 7 days. However, Ad set 6 enrolled no participants. In Ad set 7, we expanded age restriction from 18-25 to 18-50 but enrolled no additional participants. In Ad set 8, we moved up the conversion to screeners from the final Web page but this only resulted in enrolling one additional participant.

In this study, for conversion, the cost per results was *View Contents*, which ranged from US $17.64 to US $741.66. For traffic, the cost per result was *Link Clicks,* and the cost was significantly lower, which ranged from US $1.61 to US $3.07. When interpreting the performance of each Ad set, we could not conclude that the later sets (Ad set 3-7) with new features were ineffective. Ad sets were not run independently, even if they ran separately, thereby it is critical to understand that the subsequent Ad sets used pixel information from the previous sets. In fact, the new sets still let our team deliver the advertisement to the same group of Facebook users, and it is likely that we simply exhausted the pool of potential participants with the limited budget allocated to the advertisements. In essence, repeated exposure of the advertisement may not have yielded much more enrollment because those interested in the study had already clicked the advertisements and completed the screener.

Using traffic tracking, we launched 2 Ad sets from October 12 to October 26, 2017 (Table 2).

As we had been using pixel from Ad sets 1 to 8, our pixel had accumulated information regarding the participants who enrolled in our study. With this pixel, we created a *targeted audience*. This *targeted audience* was considered too specific according to the Facebook platform and estimated daily results indicated that the traffic advertisement could reach about 16 to 40 individuals. Perhaps, because of this very narrow target, the rate of enrollment was slow as we enrolled 5 participants in 10 days. However, considering that we only spent US $27.64, we observed a remarkable reduction in per enrollment cost from US $150 to US $250 to US $4.60 per participant. These new Ad sets appear to constitute a better target pool given how the pixels track more information from site interaction. Perhaps, because of this, the traffic tracking advertisements were delivered to a new audience not included in the previous 8 sets that employed conversion tracking.

**Figure 2:**
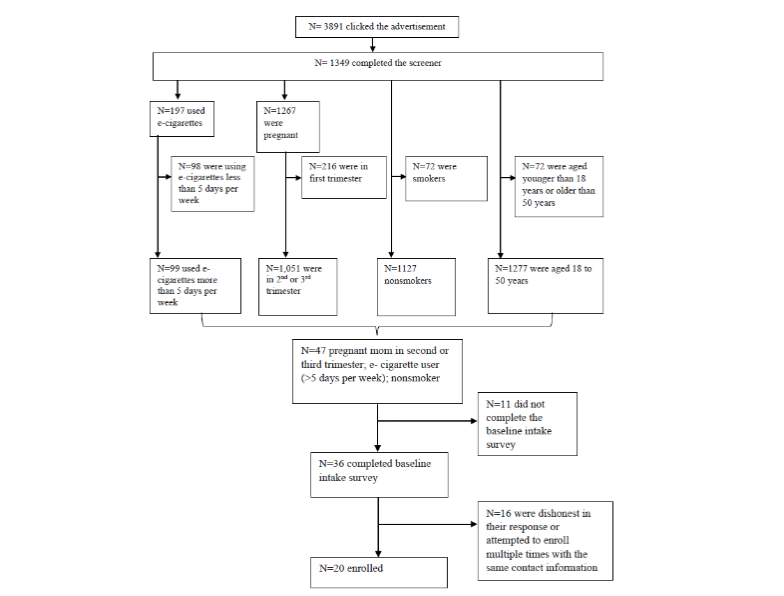
Flow diagram from the Facebook advertisements to enrollment. e-cigarette: electronic cigarette.

The flow diagram of Facebook advertisement clicks (N=3891) to final enrollment (n=20) is illustrated in Figure 2. Of 3891 Facebook users who clicked the advertisement, 34.67% (1349/3891) completed the screener. Among the 1349 participants who completed the screener, only 3.48% (47/1349) met the eligibility criteria. Among 1349 participants who completed the screener, 6.82% (92/1349) were dual users, 5.34% (72/1349) used tobacco only, 7.78% (105/1349) used e-cigarettes only, and 58.86% (794/1349) used neither tobacco nor e-cigarettes. Among the 197 e-cigarette users, 49.7% (98/197) were ineligible because of low frequency of e-cigarette use (<5 days per week). Most who completed the screener (93.92%, 1267/1349) were pregnant, 59 (4.37%, 59/1349) were not pregnant, and 23 were (1.70%, 23/1349) missing. Among those completing the screener, 1051 (77.91%, 1051/1349) were in their second or third trimester, 211 (15.64%, 211/1349) were in their first trimester, and 87 (6.45%, 87/1349) did not indicate a trimester. Taken altogether, there were 47 survey respondents, who simultaneously met all of the inclusion criteria who were then directed to the second page, *Informed Consent*, and almost all did so (97%, 46/47) with 78% (36/47) going on to complete the intake interview.

Demographic characteristics of the enrolled participants are shown in Table 3.

The enrolled participants likely had a high literacy level considering that 65% of the participants were at least *some college* or above. On average, participants were about 22 years old (mean 21.6, *SD* 2.0), with prepregnancy body mass index placing them in the overweight to obese category (mean 29.6, *SD* 6.8). On the basis of the survey about participants’ past tobacco use, 9 (36%, 9/25) smoked regular cigarettes before using e-cigarettes, 9 (36%, 9/25) used a vaping device to try to quit smoking completely, and 11 (44%, 11/25) smoked regular cigarettes before using e-cigarettes. e-cigarette: electronic cigarette

**Table 2:** Traffic per Facebook advertisement specifications and enrollment outcome.

Advertisement set (Ad set)	Lifetime spend (US $)	Duration (days)	Number of advertisements	Total cost (US $)	Total enrolled	Total screened
Ad set 9	1000.00	11	1	27.63	5	9
Ad set 10	1000.00	3	3	161.41	0	201

**Table 3:** Demographic characteristics (n=20).

Characteristics		Values
Age (years), mean (SD)		21.6 (2.0)
Prepregnancy body mass index (kg/m^2^), mean (SD)		29.6 (6.8)
**Education, n (%)**		
	High school graduate	6 (30)
	Some college	9 (45)
	College graduate	1 (5)
	Postgraduate work	1 (5)
	Do not know or prefer not to answer	3 (15)

### Relationship Management

**Figure 3:**
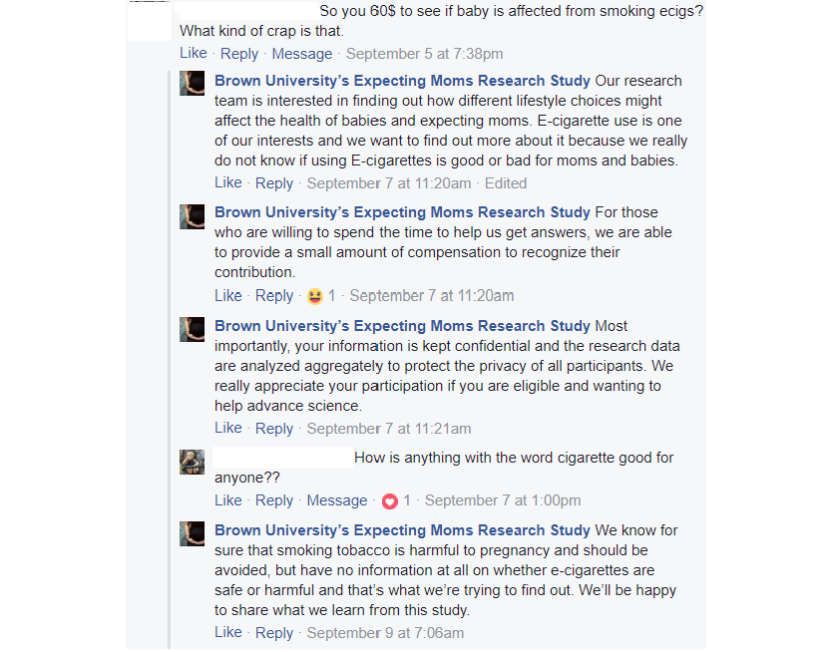
Addressing a negative comment from a Facebook user.

Studies that recruit participants via Facebook may elicit Facebook users’ questions or complaints about the study. Negative posts may influence the recruitment process as prospective respondents can see the unfavorable comments and might avoid the advertisement. We responded to negative comments from a Facebook user who later enrolled in the study. This user posted a comment on one of the advertisements in the Ad set 3 (September 4-8), questioning the ethical basis for this research on the presumption that a pregnant woman’s e-cigarettes usage would have a negative effect on the baby. We responded to the post explaining the scientific rationale and ethical ground of this research (Figure 3). 

Participants were asked for a 3-day period in which they preferred to receive the saliva sample kit—between the day after the survey and mother’s due date for her baby—and were asked to provide their address, name, and contact information. Once they submitted the survey, research staff provided a US $10 Amazon gift card via email. Among 36 participants, research staff excluded 16 participants (44%) who appeared to be dishonest in their responses, presumably to collect the incentive. For example, some participants attempted to enroll twice in the study using a different name but the same contact information. One participant claimed that she had not received the Amazon incentive after our research team had received an automated message from Amazon.com stating that she had redeemed her gift card. After these exclusions, we were able to retain 20 participants.

### Subsequent Data Collection From Participants

Of the 20 women enrolled, we were able to obtain biospecimens from 16 participants (80%), and we were able to obtain a photo of the e-cigarette device from 4 participants. A total of 10 participants provided follow-up data including their physician’s contact information. Of the 10 physicians contacted, 6 responded to our mail and provided follow-up information of the participant and the baby. Our team did not specify a period for participants in which they should submit the aforementioned data to obtain incentives.

## Discussion

### Principal Findings

Results from this study may help to inform strategies used to recruit pregnant women who use e-cigarettes but not tobacco into a study with a protocol that has multiple, demanding components. On the basis of the results of the Facebook advertisements, the lesson learned from this study regarding identifying rare populations was the importance of *targeting criteria*.

Although the advertisements recruited many pregnant mothers, it recruited few pregnant e-cigarette users. Specifically, among the 1349 participants who completed the screener, only 197 used e-cigarettes whereas almost all were pregnant. As we did not want to disclose our direct interest in e-cigarette usage because we were concerned that it could lead to false reporting to receive a financial incentive, the picture and text accompanying the advertisement did not imply that this was a focus of the research. In contrast, it was clear that the study was about pregnancy health based on the pictures and the title of the study (Figure 1). In this regard, the targeting criteria of Ad set parameters—which surreptitiously deliver the advertisements based on a Facebook user’s profile and behaviors—perhaps should have solely focused on e-cigarette behavior while forgoing other parameters (eg, *pregnancy* or *new mom*) initially. By doing so, we could have reached more e-cigarette users and then proceeded to seek those who were pregnant. The Facebook pixel function should be focused on the hardest-to-reach characteristics of the population.

A related lesson learned in this study is that a robust recruitment budget is needed to attain the desired number of participants. Simply put, a general principle of Facebook advertisements is that the more money advertisers allocate, the more people are exposed to the advertisement. Furthermore, the recruitment period must be sufficiently long to obtain the desired number of participants**.** We achieved the greatest success when allowing recruitment to remain open for weeks rather than days. This likely helps maximize the reach of the advertisement and allows the Facebook algorithm to place the advertisement in front of those that best match willing, eligible individuals. Considering the remarkable drop in the per enrollment cost from US $150 to US $250 to US $4.60 per participant, it seems reasonable to conclude that a reduced enrollment process benefitted solely from changing to traffic tracking from conversion tracking. However, while running the advertisements using conversion tracking for 3 weeks, the pixel accumulated extensive demographic information of the Facebook users who were exposed to our advertisements. With this pixel, later advertisements based on traffic tracking were able to expose the advertisements to those who were likely to enroll in this study. In other words, the remarkable drop in enrollment cost is likely attributed to both prolonged Facebook pixel exposure and choosing traffic tracking. Altogether, this suggests that the Facebook pixel could eventually identify the target population with a reasonable budget, a tracking system that suits the nature of enrollment process (ie, traffic vs conversion), and a sufficiently long recruitment period and the utility of the pixel. A third lesson learned is with regard to participant burden. We likely lost some participants because of the length of the intake assessment, which took approximately 15 to 20 min, as 10 eligible participants discontinued. In retrospect, providing a congratulatory message in the process of the survey may have been an effective method to prolong participants’ engagement with the survey. Finally, it may have been useful to obtain qualitative feedback at the end of the study.

On the contrary, the collection of biospecimens by mail was feasible as indicated by an 80% completion rate for the saliva samples (16 samples out of 20 participants). Of note, this may be generalizable to a study protocol that enrolls participant who demonstrate a certain level of commitment toward the study (eg, 20-30 min of intake survey). Saliva samples can be a rich source of biological information, and such objective assessments of individual traits can vastly enhance the scientific rigor of an internet-based study. It should be noted that only biomarkers that do not require supervised collection and are stable at room temperature can readily be incorporated into an internet-based study, and cotinine meets all of these criteria. Collecting photos via Dropbox was not as successful, as we obtained photos from only 20% of the participants. A possible explanation of this low rate is that many e-cigarette users may not have the vaping device any longer, as many may tend to quit tobacco use or go back to combustible cigarettes after delivering the baby. A more integrated survey approach in which users attach the photo directly to the survey may be less burdensome and more successful. Finally, collecting the baby’s health information from the physicians was challenging as we only obtained 60% of the physicians’ responses. Each of these components calls for evaluation of the study’s burden, financial incentives, and privacy considerations.

### Limitations

It is worth noting the possibility of sampling bias such that Facebook users may not be representative of the general population. A systematic review of this topic suggests that Facebook-recruited samples were generally representative of samples recruited through traditional methods, except that the socioeconomic status was higher among Facebook users [20]. Higher socioeconomic status among Facebook users has been also highlighted by a study that examined the representativeness of social media in Great Britain [21]. In addition, the requirements to read the consent form on the Web and complete questionnaires on the Web suggest that a relatively high literacy level, as well as high *internet literacy*, would be required for participation. Altogether, this study may have recruited those with a higher education level than the general population. Finally, to the extent of our knowledge, a Facebook Ad user has no insight into Facebook’s placement of advertisements under either traffic or conversion tracking. As such, it is impossible to evaluate the potential for bias beyond our initial target criteria. As with other types of quota sampling, conversion or traffic tracking strategies monitor the returns as they come in, which allows a researcher to promptly modify the recruitment criteria to optimize the sampling bias.

### Conclusions

Our study suggests that researchers focus the Facebook Ad set parameters to the hardest-to-reach features of the population (ie, e-cigarette usage) with other desired attributes apparent in the advertisements. It is essential to allocate a robust budget and provide an extended recruitment period for each Ad set. Researchers should minimize participant burden in the recruitment process to maximize enrollment and prepare responses or a Frequently Asked Questions document for participant retention and relationship management. Finally, saliva sample collection for cotinine analysis or other assays is feasible with careful attention to minimizing participant burden and providing the right incentive. More effort would be needed to determine how best to obtain better success in collecting photos and health information from physicians. These lessons are likely useful to others who are seeking to use social media to recruit participants who are rare in the population into studies with demanding protocols.

## Appendix

Multimedia Appendix 1 CONSORT-EHEALTH checklist (V 1.6.1).

## Abbreviations

Ad sets: advertisement sets
e-cigarette: electronic cigarette
